# Remarks about systematic reviews of diagnostic tests

**DOI:** 10.1590/S1516-31802012000500002

**Published:** 2012-11-13

**Authors:** Álvaro Najib Atallah, Andrea Puchnick, Daniel Wu, David Carlos Shigueoka, Gianni Mara Silva dos Santos, Hernani Pinto de Lemos, José Eduardo Mourão, Wagner Iared

**Affiliations:** I MD, PhD. Full Professor and Head, Discipline of Emergency Medicine and Evidence-Based Medicine, Escola Paulista de Medicina, Universidade Federal de São Paulo (EPM-Unifesp), and Director of the Brazilian Cochrane Center, São Paulo, Brazil. Scientific Director of Associação Paulista de Medicina (APM), São Paulo, Brazil.; II BSc. Professor and Coordinator of Educational and Research Support, Department of Diagnostic Imaging, Escola Paulista de Medicina, Universidade Federal de São Paulo (EPM-Unifesp), São Paulo, Brazil.; III Undergraduate Student of Medicine, Escola Paulista de Medicina, Universidade Federal de São Paulo (EPM-Unifesp), São Paulo, Brazil.; IV MD, PhD. Associate Professor, Department of Diagnostic Imaging, Escola Paulista de Medicina, Universidade Federal de São Paulo (EPM-Unifesp), São Paulo, Brazil.; V MSc. Statistician, Universidade Federal de São Paulo (Unifesp), São Paulo, Brazil.; VI MD, PhD. Research Assistant, Discipline of Emergency Medicine and Evidence-Based Medicine, Escola Paulista de Medicina, Universidade Federal de São Paulo (EPM-Unifesp), and Brazilian Cochrane Center, São Paulo, Brazil.; VII MD, PhD. Associate Professor of Radiology, Department of Medicine, Universidade Federal de São Carlos (UFSCAR), São Carlos, São Paulo, Brazil.; VIII MD, PhD. Research Assistant, Brazilian Cochrane Center, São Paulo, Brazil.

From the start of the second half of the 20^th^ century, the evolution of medicine was seen to be accelerating, and the new millennium began with a fast pace. Doctors and other health professionals cannot survive unless they are up to date with the constant changes imposed by technology, especially in big cities.

Over the last few decades, the avalanche of new drugs and modern methods of treatment has encouraged doctors to look for faster ways to follow these changes, as well as ways to verify the true efficacy of these new interventions. All of these changes led to evidence-based medicine, which has subsequently become known as evidence-based healthcare, when other healthcare professionals are included in this.

Evidence consists of nothing more than the results of assessments through scientific studies with reproducible methodological quality (for all the data described in the work). This is possible in intervention studies on high-prevalence diseases because they present large numbers of participants. However, in cases of low-prevalence diseases, the results give rise to doubts. This has led to the use of systematic reviews, since these are able to fulfill the need to evaluate intervention studies. However, this is only possible when studies are designed to have similar objectives and interventions and their methodological quality can be assessed. In a systematic review, it is desirable and often feasible to obtain the sum of statistical data from several studies, which is called meta-analysis.

More recently, the same need has arisen in the field of diagnostics. The evolution of diagnostic equipment and the emergence of new laboratory kits with promises of faster, more accurate and less invasive diagnostic methods has been widely broadcasted in the media. This directly influences patients’ opinions, and affects the people responsible for doing the examinations and governments. Patients obviously want to have access to the best examination that there is, while professionals and governments want to make sure that these new tests really are superior to the existing ones, so that the possibly high financial investment can be justified. Therefore, systematic reviews on diagnostic accuracy studies are considered to be of great relevance.

Given the lack of consensus on the most appropriate way of conducting the systematic review method, the Cochrane Collaboration, a pioneer in implementing systematic reviews on intervention studies, decided to disseminate and encourage the development of systematic reviews on accuracy studies by creating a section aimed only at reviews on diagnostic accuracy studies. This new section forms part of the RevMan (Review Manager) software, which the Cochrane Collaboration maintains in order to guide the elaboration of reviews and enable production of meta-analysis whenever possible. This brilliant initiative has caught the attention of many researchers and has encouraged them to work in this field, but just as in any other movement in its initial phase, several unresolved issues still hinder the work.

The tools for assessing the quality of individual diagnostic accuracy studies are different from those applied in intervention studies. This has generated great confusion among researchers and among editorial boards evaluating such studies. There are several published systematic reviews on accuracy studies that are full of inappropriate terms and were methodologically designed as if they were intervention studies. Furthermore, there is a lack of significant accuracy values, such as sensitivity, specificity and predictive values.

Articles cannot and should not be written for the sole purpose of having them read and evaluated by researchers and bureaucrats. The quality of tests needs to be defined, as does their performance, in terms of superiority, inferiority or resemblance in relation to preexisting tests. Also, this must be carried out honestly in presenting the data. The way to show evidence is not to present unnamed percentage indicators and let the reader do the math. All the data presented must be named, so that the conclusion of the study can be enhanced through significant and intelligible results, and not through personal opinions.

Whenever two or more studies with similar designs evaluating the accuracy of a particular test within the same patient spectrum are found in a systematic review, it is possible to obtain summary results, i.e. a meta-analysis, which must be expressed in terms of sensitivity and specificity with the respective confidence intervals. This provides the possibility of inferring additional data, such as positive and negative predictive values and likelihood ratios.

One important difference between systematic reviews on accuracy studies and on intervention studies is the quality assessment. To assess the quality of each case, Sackett et al. suggested that four questions needed to be answered: 1) Is there any blinding of the results between the index test and the standard reference? 2) Is the patient spectrum adequate? 3) Is there independence in applying the standard reference? 4) Is the standard reference applied to the entire sample?^1^ However, experience has shown that these four questions were insufficient to assess the quality of many studies.

QUADAS (Quality Assessment of Diagnostic Accuracy Studies) is a tool that was developed to assess other relevant issues.^2^ It consists of 14 questions detailing the characteristics of the patient selection (patient spectrum); partial verification bias (whether the whole sample was subjected to the standard reference); differential verification bias (whether more than one reference standard was applied); blinding of the results; reference standard and index test characteristics; and losses in the study, From this, the reporting and internal and external validity can be evaluated. However, there were still situations in which the questions of QUADAS were not applicable, and others in which further questions needed to be included.

QUADAS-2 is now available. This is a tool that adapts to the type of test and disease that is to be evaluated and consists of four key domains: 1) patient selection; 2) index test; 3) reference standard; and 4) flow and timing. Each domain assesses the risk of bias and the first, second and third domains also evaluate the study applicability. This tool basically involves drawing up a hypothetical ideal model for an accuracy test, so that a specific test can be assessed for a specific patient spectrum. Relevant questions are asked in order to compare the idealized test with each study that has been found through the search strategy.^3^

Whenever diagnostic accuracy studies under similar clinical and evaluation conditions are grouped, one concern that always comes up is the heterogeneity of the results. While heterogeneity is an exception in intervention studies, it is the rule in diagnostic accuracy studies.^4^ The source of the heterogeneity is not always clear, although it is possible that the patient spectrum at different research sites may be the main factor in most cases. Nevertheless, these sources must of course be evaluated individually. In systematic reviews on intervention studies, one or more studies with very heterogeneous results may be excluded from the meta-analysis. Because the patient spectrum, target condition, evaluated test and reference standard are the same in diagnostic accuracy studies, this kind of exclusion should not take place in systematic reviews on such studies.

For this reason, choosing the best way to mathematically group the results from several accuracy studies has led to a dead end. For non-heterogeneous studies, because there is no randomization in quality accuracy studies, grouping them is a valid option for performing meta-analysis. However, whenever heterogeneity is present, this factor must be taken into account. Comparison of different individual tests in a systematic review can be done visually using a receiver-operating characteristic (ROC) curve, or using a forest plot in situations in which sensitivity and specificity values with their respective confidence intervals are available.^4^ The Cochrane Collaboration has suggested that the hierarchical summary receiver operating characteristic (HSROC) model^5^ or the bivariate model^6^ should be used to obtain summary estimates of sensitivity and specificity with their respective confidence intervals.

We are looking forward to the promised update from RevMan for accuracy studies in order to facilitate fulfillment and standardization of the results and conclusions among authors.
